# Osteoporosis recovery in severe anorexia nervosa: a case report

**DOI:** 10.1186/s40337-019-0269-8

**Published:** 2019-11-08

**Authors:** Pratibha Anand, Philip S. Mehler

**Affiliations:** 1 University of Colorado School of Medicine, Aurora, Colorado USA; 2 Eating Recovery Center of Denver, Denver, Colorado USA; 3 ACUTE, Denver Health, Denver, Colorado USA

**Keywords:** Anorexia nervosa, Osteoporosis, Teriparatide, Denosumab, Hormonal replacement therapy, Supplementation

## Abstract

**Background:**

Osteoporosis represents a common and severe complication in patients with anorexia nervosa (AN) that normally persists despite weight restoration and the resumption of regular menses. The condition may result in significant pain, injury, and disability.

**Case presentation:**

We report the only published case of a complete return to normal bone density following many years of severe osteoporosis in a severely malnourished patient with AN. We describe a patient with severe and enduring AN whose osteoporosis, with resultant fractures, was completely reversed. Available patient records, imaging, and laboratory data were evaluated.

**Conclusions:**

This case represents a common yet often improperly treated complication of AN. It demonstrates the potential important clinical role that targeted medicines coupled with a multifaceted supplementation and lifestyle interventions, may have for some very malnourished patients with AN. Medications, in the treatment of osteoporosis in patients with severe AN, may decrease fracture risk, enhance overall bone density, and contribute to an improved quality of life.

## Introduction

Osteoporosis represents a common and severe medical complication in patients with anorexia nervosa (AN). Osteoporosis is a skeletal disease characterized by reduced bone density and microarchitectural deterioration of normally mineralized bone. The decreased bone mass results in fragility and diminished mechanical strength, leading to an increased fracture risk [1]. It is a silent disease that frequently goes undiagnosed or delayed in diagnosis until a patient experiences fractures following minimal or, in certain cases, no trauma [2].

Osteoporosis is generally stratified into three subcategories. Osteopenia is characterized by bone mineral density that is 1 to 2.5 standard deviations below the young adult mean value. Osteoporosis is defined clinically as radiographically assessed bone mineral densit that is at least 2.5 standard deviations below the bone mineral density observed in a healthy young adult population. Finally, severe or established osteoporosis is characterized by bone mineral density that is greater than 2.5 standard deviations below the young adult mean value with one or more concomitant fragility fractures [3].

Bone loss in patients with AN is multifactorial: it a result of a deficiency in sex hormones and other endocrine factors as well as direct effects of undernutrition. The loss of bone mineral density (BMD) also takes place rapidly, often occurring within 6 months of disease onset [4] and persisting even after weight recovery [5]. Specific causes of low BMD in AN, notwithstanding the typically young age of patients with AN, include hypoestrogenism, hypoandrogenism, malnutrition, reduced lean body mass, and hypercortisolemia. Also, insulin-like growth factor 1 (IGF-1), a bone trophic factor, is decreased in patients with AN in spite of elevated growth hormone (GH) levels, as a result of an acquired GH-resistant state. Increased ghrelin and peptide YY levels may also play a role [6, 7].

To our knowledge, no cases describing the completely successful medicinal treatment and reversal of severe osteoporosis in patients with extreme AN have been reported. Furthermore, while treatments are available for osteoporosis, no cure exists at this time [8]. We describe a case in which a patient with severe anorexia nervosa, and osteoporosis dating back nearly 20 years to pre-adolescence, ultimately achieved a return to normal bone density in her late 20s.This case report may facilitate further research into the timely utility of teriparatide and other medicinal interventions in the treatment of selective cases of severe AN with osteoporosis.

## Case history

This case involves a 27-year-old female with a 17-year history of severe AN who was first diagnosed with osteoporosis at age 14. Her eating disorder began at the age of 10 when she first started engaging in dietary restriction and compulsive exercise subsequent to an episode of acute food poisoning. Since that time, she has been medically hospitalized close to 100 times and has received specialized inpatient and residential eating disorder treatment at times spanning several weeks to several months. The patient’s lowest adult weight was 43 pounds (162 cm/ 64 in., BMI 7.4) and her highest adult weight was 112 pounds (BMI 19.2).

Osteoporosis was first diagnosed in the patient’s hip and spine at the age of 14. Since that time, the patient has received bone density (DXA) scans every 1–2 years, with worsening bone density up until age 25, in spite of receiving various treatments for her osteoporosis. She took 1000 mg of calcium carbonate supplementation between the ages of 15 and 17. At the age of 18, she commenced weekly alendronate sodium treatment that was discontinued after 3 months due to complaints of nausea. Between the ages of 19 and 22, the patient received annual IV zolendronic acid treatment with continued ongoing decline in her bone mineral density.

The patient has never had a spontaneous menarche. She began combination oral birth control pills (drospirenone 3 mg and ethinyl estradiol 0.02 mg) between the ages of 19 and 22. Since the age of 23, the patient has been using biweekly transdermal estradiol patches (0.075 mg/ day) and takes 200 mg of bioidentical micronized progesterone for the first 12 days of each month. She has menstruated regularly each month since beginning her transdermal estradiol and progesterone treatment.

At the age of 23, the patient commenced a regime of biweekly strength conditioning for approximately 45 min per session. She eliminated caffeine from her diet and consumed adequate calcium each day from a combination of diet and supplementation. The patient’s 25-hydroxy vitamin D level at this time was low at 19 nmol/L and showed minimal response to supplementation until weekly doses of 50,000 IU of vitamin D were given for 6 weeks. The patient then maintained her vitamin D levels between 40 and 50 nmol/L while taking 10,000 IU of vitamin D daily.

At the age of 23, the patient also experienced a spontaneous stress fracture on her fifth left metatarsal. In spite of adhering to treatment recommendations, it took 12 weeks for the patient to be able to walk again without pain. The patient experienced another stress fracture a year later in the same site as a result of excessive walking and jogging.

In July 2015 and December 2015, at the age of 24 and shortly after her 25th birthday respectively, the patient first received denosumab. Her weight at time ranged between 52 and 78 pounds (BMI 8.9–13.4). Her DXA scan from that year showed her first ever improvements in bone mineral density (T scores between − 2.3 and − 1.2) since being diagnosed with osteoporosis.

In May 2016, the patient experienced a spontaneous first rib fracture that was diagnosed coincidently when obtaining an x-ray for a dislocated shoulder. One year later, in June 2016, the patient also began a treatment course of daily teriparatide injections lasting 2 years. DXA scan results obtained in December 2016 are as follows:

**Table:** 

Site	BMD (g/cm^2^)	T-score (STD)	Z-score (STD)
L1-L4	0.962	−1.8	− 1.0
Left Femoral neck	0.881	−1.1	−0.6
Left Femur Total	0.814	−1.5	−0.9
Right Femoral Neck	0.909	−0.9	−0.4
Right Femur Total	0.814	−1.5	−0.9

During the course of her daily teriparatide treatment, the patient’s weight ranged between 58 and 84 pounds (BMI 10.0–14.4). She reported no side effects either during or following this course of treatment.

The patient’s most recent DXA scan results from August 2018 demonstrated bone density values within the normative range for age and gender:

**Table:** 

Site	BMD (g/cm^2^)	T-score (STD)	Z-score (STD)
L1-L4	1.019	−1.3	− 0.3
Left Femoral neck	0.934	−0.8	−0.0
Left Femur Total	0.881	−1.0	−0.2
Right Femoral Neck	0.934	−0.7	−0.0
Right Femur Total	0.867	−1.1	−0.3

Notably, her left femur total showed an increase of 0.067 g/cm2 in density, a change of 8.2%. Her right total femur showed an increase of 0.053 g/cm2 in density, a change of 6.5%.

## Discussion

At present, there is no standard treatment recommendation for patients with severe AN who have osteoporosis. Weight restoration and a resumption of menses remain the mainstays of treatment. However, a longitudinal study adolescent AN showed enduring adverse impact on bone health even 10 years following the normalization of body weight [9]. Previous studies have likewise reported persistent, long-term increased bone fracture risk many years following the initial diagnosis of AN [10]. Furthermore, given the often chronic and refractory nature of the illness and the long-term increased fracture risk in this young population, the aforementioned case provides a call for further empirical investigations of the use of denosumab and teriparatide in patients with AN who have osteoporosis.

This patient’s case appears to align with studies that suggest that teriparatide increases BMD and decreases the risk of vertebral fracture and hip fracture due to its anabolic effects on bone new bone formation [11]. Teriparatide has also been shown to increase spine BMD in patients with AN after only 6 months of therapy [12]. A case report of the use on denosumab in an osteoporotic patient with AN likewise demonstrated improved BMD with this treatment [13]. These findings also support data drawn from the 2-year DATA study [14] and the cumulative 4-year outcomes from its extension study, DATA-Switch [15], that show that the combination of teriparatide and denosumab outperformed either drug individually.

## Conclusion

In summary, rigorous randomized controlled studies continue to be needed and warranted in determining the efficacy of newer and traditional osteoporosis interventions and management in anorexia nervosa and across other populations given the high prevalence of low BMD in the population [16]. This case report provides a modicum of optimism that, with judicious use of medications and close monitoring of timely DXA scans, improvements in bone health can be achieved.

## Data Availability

Data sharing not applicable to this article as no datasets were generated or analysed during the current study.

## Abbreviations

AN: Anorexia nervosa
BMD: Bone mineral density
BMI: Body mass index
DXA: Bone densitometry/ dual-energy x-ray absorptiometry
IU: International units
